# Simple prediction of COVID-19 convalescent plasma units with high levels of neutralization antibodies

**DOI:** 10.1186/s12985-023-02007-0

**Published:** 2023-03-27

**Authors:** Katerina Jazbec, Mojca Jež, Klemen Žiberna, Polonca Mali, Živa Ramšak, Urška Rahne Potokar, Zdravko Kvrzić, Maja Černilec, Melita Gracar, Marjana Šprohar, Petra Jovanovič, Sonja Vuletić, Primož Rožman

**Affiliations:** 1 Blood Transfusion Centre of Slovenia, Šlajmerjeva 6, Ljubljana, 1000 Slovenia; 2 NIB-National Institute of Biology, Ljubljana, Slovenia

**Keywords:** COVID-19 convalescent plasma, Anti-SARS-CoV-2 antibodies, Neutralization assay, Antibody titer, Hyperimmune plasma donors

## Abstract

**Background:**

Hyperimmune convalescent COVID-19 plasma (CCP) containing anti-SARS-CoV-2 neutralizing antibodies (NAbs) was proposed as a therapeutic option for patients early in the new coronavirus disease pandemic. The efficacy of this therapy depends on the quantity of neutralizing antibodies (NAbs) in the CCP units, with titers ≥ 1:160 being recommended. The standard neutralizing tests (NTs) used for determining appropriate CCP donors are technically demanding and expensive and take several days. We explored whether they could be replaced by high-throughput serology tests and a set of available clinical data.

**Methods:**

Our study included 1302 CCP donors after PCR-confirmed COVID-19 infection. To predict donors with high NAb titers, we built four (4) multiple logistic regression models evaluating the relationships of demographic data, COVID-19 symptoms, results of various serological testing, the period between disease and donation, and COVID-19 vaccination status.

**Results:**

The analysis of the four models showed that the chemiluminescent microparticle assay (CMIA) for the quantitative determination of IgG Abs to the RBD of the S1 subunit of the SARS-CoV-2 spike protein was enough to predict the CCP units with a high NAb titer. CCP donors with respective results > 850 BAU/ml SARS-CoV-2 IgG had a high probability of attaining sufficient NAb titers. Including additional variables such as donor demographics, clinical symptoms, or time of donation into a particular predictive model did not significantly increase its sensitivity and specificity.

**Conclusion:**

A simple quantitative serological determination of anti-SARS-CoV-2 antibodies alone is satisfactory for recruiting CCP donors with high titer NAbs.

**Supplementary Information:**

The online version contains supplementary material available at 10.1186/s12985-023-02007-0.

## Introduction

The hyperimmune convalescent COVID-19 plasma (CCP) with anti-SARS-CoV-2 antibodies became an appealing source of therapy early in the COVID-19 pandemic. Millions of CCP units were collected in the following two years and became available for controlled prospective clinical studies. The promising prospect was that the antibodies present in CCP units could neutralize the SARS-CoV-2 virus contributing to its clearance from the patient [1, 2].

During COVID-19 infection, the immune system generates various classes of anti-SARS-CoV-2 antibodies (Abs) that are polyspecific and polyclonal. The early IgM and IgA Abs peak between day 16 to day 30 after symptom onset and disappear 1–2 months later, whereas the IgG Abs persist much longer. In most infected patients, anti-SARS-CoV-2 IgG seropositivity lasts for at least six months after infection [3, 4], which renders them potential donors of CCP.

Several initial studies on low numbers of patients reported a beneficial effect of CCP treatment with no serious side effects [5–7]. The initial studies had some drawbacks, such as a lack of suitable control groups, variability of therapeutic approaches, concomitant pathology, and poor recording of adverse reactions. Probably the most significant failure was the lack of uniform timing and dosing of CCP therapy. However, the results gave specific common knowledge that only high titer plasma should be used, that it should be used within 72 h of the symptom appearance, and that only selected groups of patients are legible for CCP therapy, such as mildly affected patients, and especially the ones with immune deficiencies [8, 9]. However, several parallel studies haven’t yielded such booming therapeutic results [10, 11]. At least two meta-analyses did not provide evidence of a reduction in mortality or any benefit for other clinical outcomes [12, 13]. Another subset of patients who received CCP within 72 h of symptoms onset did not show significant improvement [14], even when two CCP units with high antibody titer were administered and the patients had some benefit. International trials such as RECOVERY and REMAP-CAP demonstrated a low likelihood of improvement in organ support-free days and mortality [15, 16]. Also, later on in 2022, several groups failed to report the mortality benefit of CCP treatment in patients with mild disease [17] as well as with severe disease [18–22], which led to the most recent AABB expert panel’s clinical practice guidelines recommending CCP therapy only for outpatients with COVID-19 who are at high risk for disease progression, for hospitalized patients with moderate or severe disease, and for the patients with COVID-19 who do not have SARS-CoV-2 antibodies at admission or with preexisting immunosuppression. Besides, the CCP should not be used prophylactically for uninfected persons with close contact exposure and should be transfused with high neutralizing titers to infected patients early after symptom onset [23].

Therefore, it was somehow surprising that in 2022, the benefit of early therapy was again confirmed in several larger cohorts. Sullivan et al. reported that early administration of high titer CCP reduced hospitalizations by more than 50% [24]. Similarly, Sanz et al. reported that transfusion of CCP caused a significant reduction in the 30-day mortality rate, suggesting that CCP can still be helpful in selected patients and calling for further studies before withdrawing CCP from the COVID-19 therapeutic armamentarium [25]. Franchini et al. even urge their colleagues to review the available CCP efficacy data and incorporate its use in the treatment of the vulnerable population [26]. Many other studies reported favorable responses to CCP treatment, too [27–30].

During 2020 and 2021, we collected approximately 4000 hyperimmune CCP units from voluntary blood donors. The entering criterion was a recovery after PCR-positive COVID-19 disease. CCP donors are often selected based upon their neutralizing antibody (NAb) count, which is assessed by a plaque reduction neutralization test (NT) that needs a feasible isolate, replication-competent cell lines, and qualified staff [31]. Our chosen threshold for effective CCP units was the NAb titer ≥ 1:160, based on the references stating that people with NAb titer ≥ 1:160 can be protected from the SARS-CoV-2 infection [32, 33]. Since the standard NTs are technically demanding, expensive, and require biosafety level 3 containment, we looked for a simple prediction method for acquiring CCP units with corresponding therapeutic efficiency based on the demographic, clinical, and serological status of the convalescent donors. We intended to identify the key features that would predict the high NT values. These results would be applicable not only for determining appropriate hyperimmune CCP but also to prevent and control COVID-19 infection and optimize vaccine doses [34].

## Methods

### The study design

For this retrospective cohort study, we analyzed 1302 CCP units collected from convalescent donors that donated plasma between June 2020 and August 2021 and divided them into a group with low NT values (< 160) and a group with high NT values (≥ 160). With univariate analysis, we determined the demographic and laboratory parameters that differed in each group and were therefore considered informative. We created four different models, which included different potentially informative parameters such as serology assays, demographic data, and the post-COVID-19 period of collection.

### Ethics

The study was approved by the National Medical Ethics Committee of the Republic of Slovenia (0120–241/2020-11, from 7.12.2020; 0120–241/2020/14, from 17.5.2021; 0120–241/2020-8, from 18.6.2020).

### Convalescent plasma donors

All CCP donors met the criteria for normal blood or plasma donation. Only the first donations from 1302 CCP donors (400 females and 902 males)aged between 18 and 65 years (mean 43.9 ± 0.3 years) with a history of polymerase chain reaction (PCR) confirmed SARS-CoV-2 infection in nasopharyngeal swabs were included. Out of these, 86% of participants reported mild symptoms, 14% reported moderate to severe symptoms, with only 6 participants requiring hospital treatment. CCP units were donated 63.0 days [IQR 43.0-121.0 days] after confirmed SARS-CoV-2 infection. Since the vaccination in our region started after January 2021, 235 convalescent donors have also received the vaccination before the first CPP collection. Basic donor information is presented in Table 2.

### Serological testing

Two semi-quantitative and one quantitative serological test were used to detect anti-SARS-CoV-2 antibodies: *(i)* Wantai SARS-CoV-2 Ab ELISA, an enzyme-linked immunosorbent assay for the qualitative detection of total IgG and IgM antibodies to the RBD of *S*ARS-CoV-2 spike protein that was performed on 285 samples; *(ii)* Abbott SARS-CoV-2 IgG assay, a chemiluminescent microparticle assay (CMIA) for the qualitative detection of IgG Abs to the nucleocapsid protein that was performed on 569 samples; and *(iii)* Abbott SARS-CoV-2 IgG II Quant, second-generation CMIA for the quantitative determination of IgG Abs to the RBD of the S1 subunit of the SARS-CoV-2 spike protein, including the neutralizing Abs that was performed on 844 samples. All tests were performed according to the manufacturers’ instructions.

### Neutralizing antibody testing

For neutralizing antibody testing, a standard live SARS-CoV-2 microneutralization assay was used [35]. Briefly, two-fold serial dilutions of CCP from 1:10 to 1:1280 were prepared and mixed with a viral solution containing 100 TCID50 (TCID50–50% tissue culture infective dose) and incubated. Virus lineage B.1.1 (G614) was used. 10,000 Vero E6 cells per well of 96-well plate were preseeded 24 h before the experiment. After incubation, the virus/CCP mixture was added to Vero E6 cells into a 96-well plate and incubated for five days at 37 °C. The assay readout was the cytopathic effect, and the assay cut-off titer was < 1:20. Neutralization test was performed at the Institute of Microbiology and Immunology, Faculty of Medicine, University of Ljubljana.

### Statistics

The analysis was performed on the COVID-19 dataset from September 2020 to August 2021. A subset table was created using only the first measurement for every donor (some donors have donated plasma multiple times) and only using data where NT measurement was obtained. This selection yielded a total of 1302 samples used for this analysis.

Data in the analysis are presented as the mean and standard error of the mean for normally distributed data, median and interquartile range (IQR) for non-parametric data, and as the count and the percentage for binary data. Statistical comparison between the two groups was performed using a Student’s t-test for normally distributed data, a Mann-Whitney U test for non-parametric data, and Fisher exact test for binary data.

Four different multiple logistic regression models were created to analyze the relative importance of selected features in predicting high neutralization test values. The dataset was split into the train set (70% of the data) that was used to fit the model. The test (validation) set (30%) was used to assess the model’s prediction performance. A receiver operating characteristics (ROC) curve was used to compare the sensitivity and specificity of different models.

The analysis was performed using Python 3.8, NumPy 1.19, Pandas 1.1, SciPy 1.7, SciKit Learn 0.23, and Statsmodels 0.12.

## Results

We compared the groups with low NAb titers and high NAb titers. The first important difference between the groups was the COVID-19 vaccination. More than 44% of samples in the high NT titer were from donors who were vaccinated against COVID-19 and also recovered from the COVID-19 infection (compared to 1.7% in the low NT titer group) (Table S1). Amongst the samples from the vaccinated donors (235 of 1302 total samples, Table S2), 94.5% of vaccinated ones were in the high NT titer group compared to 5.5% in the low NT titer group (Table 1).

**Table 1:** Unvaccinated donor’s characteristics in low and high NT titer groups

	Low NT titer (< 1:160)	High NT titer (≥ 1:160)	p-value
**Demographic parameters**			
Gender - female	263 (34.3%) (N = 766)	84 (27.9%) (N = 301)	0.050
Age (years)	42.4 ± 0.4 (N = 766)	47.4 ± 0.6 (N = 301)	< 0.001
Body weight (kg)	85.2 ± 0.7 (N = 637)	90.3 ± 1.1 (N = 199)	< 0.001
Height (cm)	175.8 ± 0.3 (N = 634)	176.0 ± 0.6 (N = 196)	0.759
Body mass index (kg/m2)	27.4 ± 0.2 (N = 633)	29.1 ± 0.3 (N = 196)	< 0.001
**Blood groups and total IgG**			
Blood group 0	252 (32.9%) (N = 766)	106 (35.2%) (N = 301)	0.472
Blood group A	314 (41.0%) (N = 766)	127 (42.2%) (N = 301)	0.730
Blood group B	134 (17.5%) (N = 766)	48 (15.9%) (N = 301)	0.588
Blood group AB	66 (8.6%) (N = 766)	20 (6.6%) (N = 301)	0.319
Rh(D) factor	569 (81.8%) (N = 696)	204 (80.0%) (N = 255)	0.574
Total IgG (AU/ml)	10.4 [9.1–11.9] (N = 732)	10.6 [9.4–11.9] (N = 280)	0.143
**Serological testing**			
Wantai semi-quantitative SARS-CoV-2 Ab test (index S/C)*	17.9 [11.2–19.3] (N = 233)	19.6 [18.6–20.5] (N = 52)	< 0.001
Abbott semi-quantitative SARS-CoV-2 Ab test (index S/C)*	4.32 [2.62-6.0] (N = 455)	6.94 [5.62–7.88] (N = 114)	< 0.001
Abbott quantitative SARS-CoV-2 Ab test (BAU/ml)	187 [141–282] (N = 385)	498 [310–1019] (N = 224)	< 0.001
Neutralization test (titer)	31.2 [30.3–32.1] (N = 766)	300.0 [288.2-312.4] (N = 301)	< 0.001
**Timeline**			
Days after start of COVID-19 symptoms	53.0 [41.0-79.2] (N = 712)	52.5 [40.0-79.8] (N = 254)	0.822
0–60 days after start of COVID-19 symptoms	418 (59.2%) (N = 706)	150 (61.2%) (N = 245)	0.597
60–120 days after start of COVID-19 symptoms	221 (31.3%) (N = 706)	76 (31.0%) (N = 245)	1.000
120–180 days after start of COVID-19 symptoms	67 (9.5%) (N = 706)	19 (7.8%) (N = 245)	0.518
**Symptoms**			
Hospitalization	4 (1.0%) (N = 335)	1 (1.0%) (N = 122)	1.000
Fever	191 (58.0%) (N = 329)	88 (71.0%) (N = 124)	0.013
Maximum body temperature (°C)	38.0 [37.5–38.5] (N = 461)	38.5 [37.8–39.0] (N = 189)	0.001
Number of days with fever	2.0 [1.0–4.0] (N = 559)	4.0 [2.0–8.0] (N = 218)	< 0.001
Cough	106 (32.0%) (N = 329)	60 (48.0%) (N = 124)	0.002
Anosmia	209 (63.0%) (N = 330)	58 (47.0%) (N = 124)	0.002
Myalgia	146 (44.0%) (N = 330)	60 (48.0%) (N = 124)	0.460
Dyspnea	34 (10.0%) (N = 329)	25 (20.0%) (N = 123)	0.007
Fatigue	168 (51.0%) (N = 329)	63 (51.0%) (N = 124)	1.000
Headache	108 (33.0%) (N = 327)	36 (29.0%) (N = 123)	0.497

Notes: All data was not available for every plasma donor. The N represents the total number of samples for which the data was available for a particular parameter

*Index S/C - signal/cut-off index

In order to depict which other parameters (besides vaccination status) could also be different between the low and high NAb titer groups, we separately analyzed the samples from unvaccinated donors (1067 of 1302 total samples). Table 1 presents how basic demographic features, blood types, SARS-CoV-2 antibody testing, and COVID-19 symptoms differ between unvaccinated donors in the low and high NAb titer groups.

The unvaccinated donors in the high NAb titer group were older (p < 0.001), and had greater body weight and body mass index (p < 0.001) compared to donors in the low NAb titer group. There were no differences between the blood group types, the total IgG value, and the duration between the start of COVID-19 and the date of plasma collection. Comparing the clinical presentation of COVID-19 between the groups, we note that the higher proportion of donors in the high NAb titer group had fever (p = 0.01), higher maximum temperature (p = 0.001), and longer symptom duration (p < 0.001). Furthermore, a higher proportion of donors in the high NAb titer group also had cough (p = 0.002), anosmia (p = 0.002), and dyspnea (p = 0.007). There were no differences in the number of hospitalizations, frequency of myalgia, and fatigue between the donors in the two groups.

Unsurprisingly, the most important features differentiating the low and high NAb titer group samples are serological measurements of SARS-CoV-2 antibodies (p < 0.001). However, there are differences between different commercial tests. The difference in medians between low NT and high NT titer groups using the Wantai semi-quantitative S/C index was 1.7 or 9%. The difference in medians between the groups using Abbott semi-quantitative S/C index was 2.6 or 61%. And, finally, the difference in medians between the groups using Abbot quantitative test was 311 BAU/mL or 166%.

**Fig. 1:**
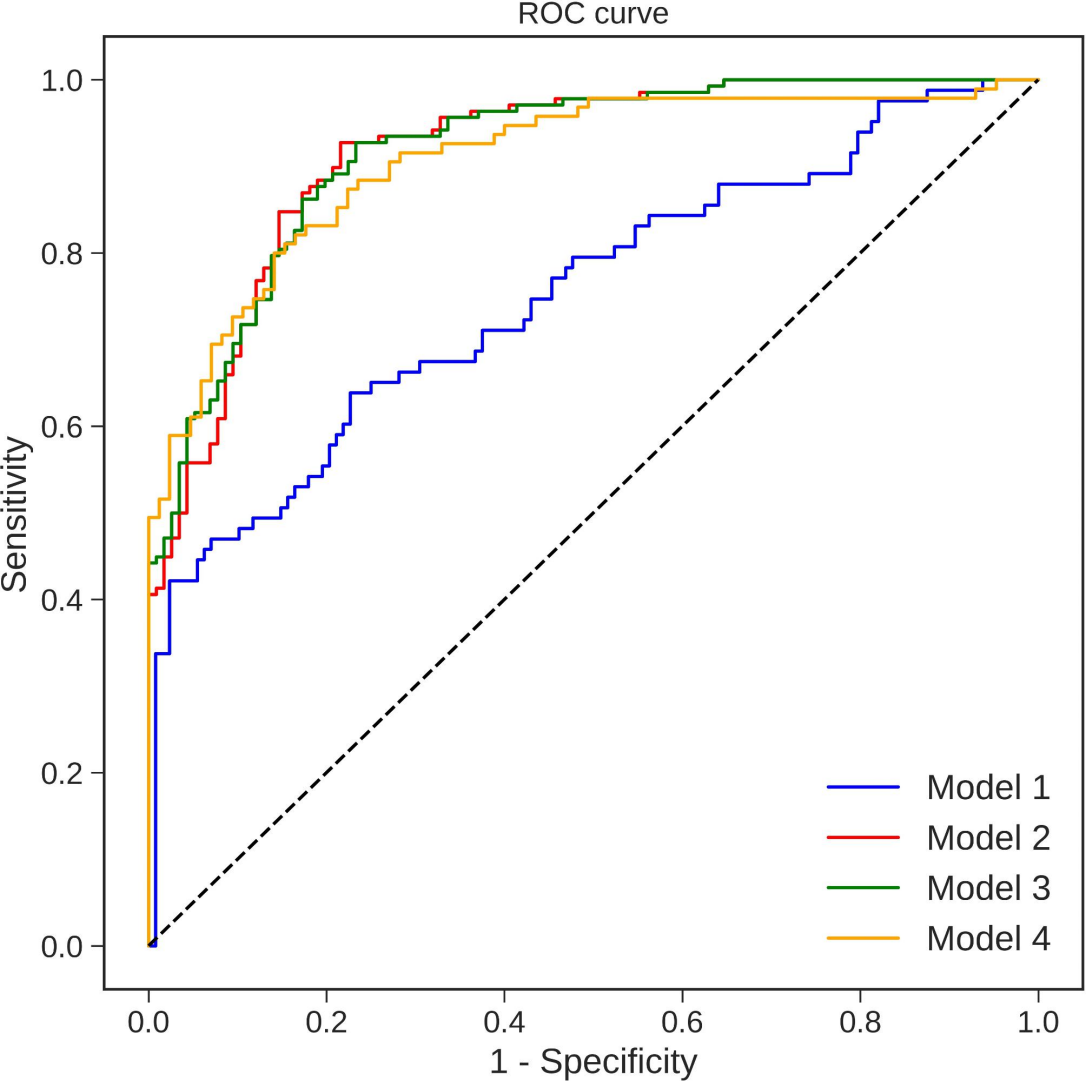
The receiver operating characteristic (ROC) curve of four different multiple logistic regression models (defined in Table 3) predicting a high NT titer group. Model 1 was created with demographic and clinical variables, Model 2 consists of Abbott quantitative SARS-CoV-2 Ab test only, the Model 3 uses Abbott quantitative SARS-CoV-2 Ab test variable and vaccination status, and the Model 4 includes all variables

Four different multiple logistic regression models were created (Table 3) to assess which parameter or combination of parameters has the most significant predictive power for choosing CCP donors with high SARS-CoV-2 NAb titer (≥ 160). The first multiple logistic regression model (Model 1) contained seven demographic and clinical variables and had relatively poor performance, with 0.75 ROC AUC (area under the ROC curve), 0.65 sensitivity, and 0.74 specificity at the optimal cut-off threshold (Fig. 1).

The second model (Model 2), consisting of the Abbott SARS-CoV-2 IgG II Quant test only, performed better than the previous model and produced a 0.83 ROC AUC with 0.80 sensitivity and 0.86 specificity at the optimal cut-off threshold. The variable Abbott SARS-CoV-2 IgG II Quant test was highly significant (p < 0.001) in this model. An increase of 100 BAU/mL results in a 1.5x increased probability of having a high NT titer.

Adding additional variables to the Abbott SARS-CoV-2 IgG II Quant test resulted in minor improvements in predicting high NT titers over the previous model. Model 3 incorporated the Abbott SARS-CoV-2 IgG II Quant test and vaccination variables and produced a 0.92 ROC AUC with 0.80 sensitivity and 0.86 specificity at the optimal threshold. Both variables were statistically significant (p < 0.001 and p = 0.02, respectively). The odds ratio (OR) for 100 BAU/mL of variable Abbott SARS-CoV-2 IgG II Quant test was 1.44, and the OR for being vaccinated was 2.64.

Finally, Model 4 included all previously selected variables and resulted in 0.91 ROC AUC with 0.73 sensitivity and 0.89 specificity at the optimal threshold. Only the Abbott SARS-CoV-2 IgG II Quant test variable and vaccination status were statistically significant (p < 0.001 and p = 0.004 respectively) in this model with OR of 1.38 and 5.66, respectively.

**Fig. 2:**
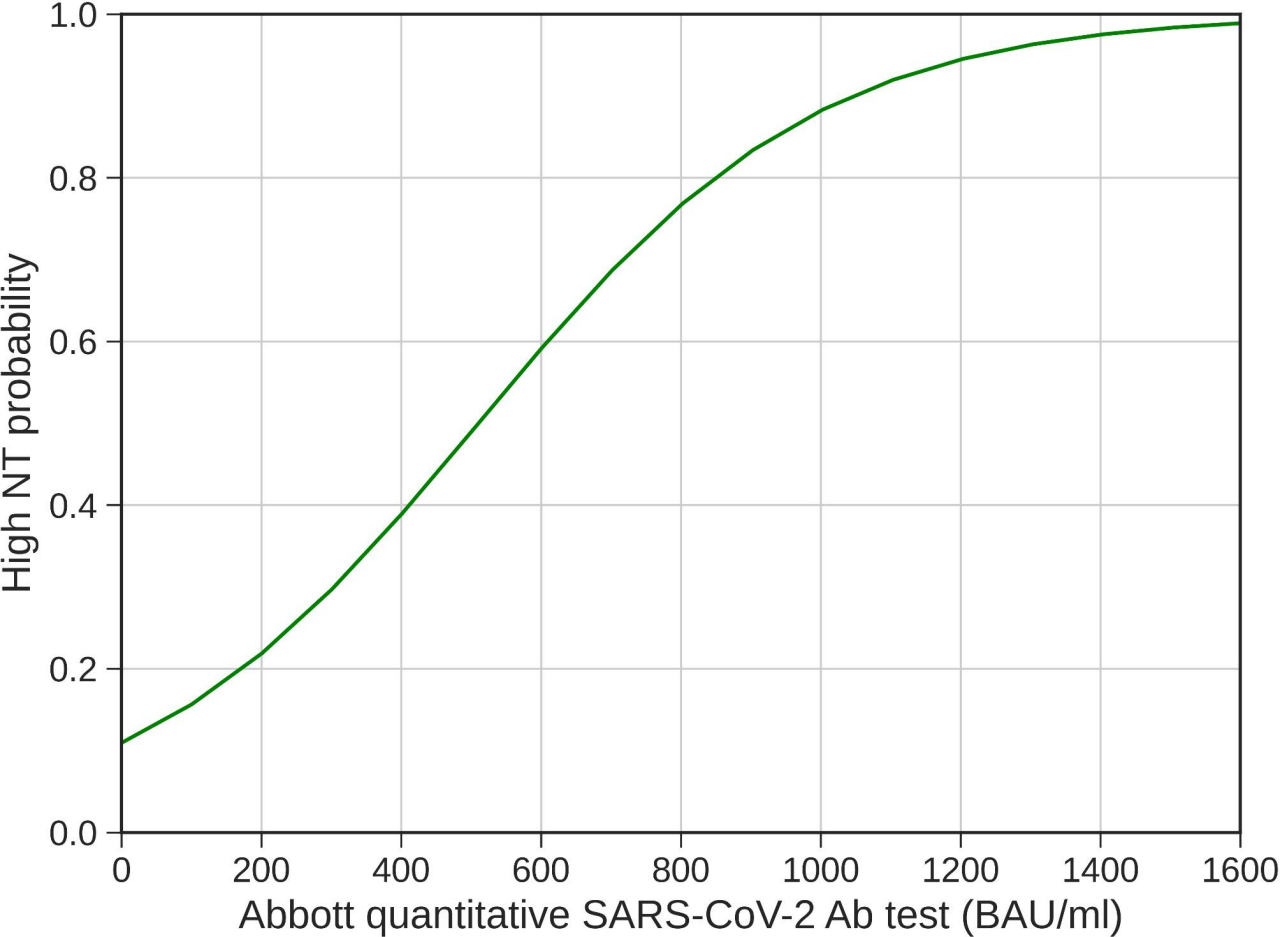
The modeled relationship between the Abbot quantitative SARS-CoV-2 Ab test and high NAb titer probability. The probability of a high NT titer is obtained from Model 2 (as defined in Table 3)

Even though the univariate analysis depicted differences in many variables when comparing the low NAb titer group with the high NAb titer group (Table 2), their inclusion into the predictive model only minimally improved its predictive power. Besides, the results of the simplest model, i.e., Model 2, are also very informative. The CCP donors with SARS-CoV-2 IgG values above 850 BAU/ml had an 80% probability of having high NT (Fig. 2). Altogether, the simplest model (Model 2) seems to be sufficient for a good prediction of high Nab titers in CCP donors, although it only relied on one variable, i.e., the quantitative serological chemiluminescent microparticle assay (CMIA) test.

**Table 2:** Number of vaccinated and unvaccinated donors with low or high neutralizing antibody titers

	Low NAb titer (< 1:160)	High NAb titer (≥ 1:160)
Unvaccinated	766 (71.8%)	301 (28.2%)
Vaccinated	13 (5.5%)	222 (94.5%)

Notes: Vaccinated donors were more likely to have a high NT titer than unvaccinated CCP donors (p < 0.001)

**Table 3:** Different multiple logistic regression models predict whether a donor will yield a high NT titer

	Log OR (95% CI)	OR (95% CI)	p-value
**Model 1**			
N training = 491, N evaluation = 211			
Gender (female)	0.14 [-0.49-0.78]	1.15 [0.61–2.17]	0.66
Age (years)	0.02 [0.0-0.04]	1.02 [1.00-1.04]	0.07
Body weight (kg)	-0.15 [-0.32-0.02]	0.86 [0.72–1.02]	0.09
Height (cm)	0.14 [-0.03-0.32]	1.16 [0.97–1.38]	0.11
Body mass index (kg/m2)	0.46 [-0.07-1]	1.59 [0.93–2.71]	0.09
Days after start of COVID-19 symptoms	0.02 [0.01–0.02]	1.02 [1.01–1.02]	< 0.001
COVID-19 symptoms duration (days)	0.01 [-0.02-0.03]	1.01 [0.98–1.03]	0.53
**Model 2 (univariate logistic regression)**			
N training = 590, N evaluation = 254			
Abbott quantitative SARS-CoV-2 Ab test (100 BAU/ml)	0.41 [0.33–0.50]	1.51 [1.38–1.64]	< 0.001
**Model 3**			
N training = 590, N evaluation = 254			
Abbott quantitative SARS-CoV-2 Ab test (100 BAU/ml)	0.37 [0.28–0.46]	1.44 [1.32–1.58]	< 0.001
Vaccination	0.97 [0.14–1.8]	2.64 [1.15–6.04]	0.02
**Model 4**			
N training = 420, N evaluation = 180			
Gender (female)	-0.53 [-1.43-0.36]	0.59 [0.24–1.44]	0.24
Age (years)	0.00 [-0.02-0.03]	1.00 [0.98–1.03]	0.77
Body weight (kg)	-0.23 [-0.47-0.02]	0.80 [0.62–1.02]	0.07
Height (cm)	0.19 [-0.06-0.44]	1.21 [0.94–1.56]	0.14
Body mass index (kg/m2)	0.7 [-0.05-1.45]	2.01 [0.95–4.27]	0.07
Days after start of COVID-19 symptoms	0.00 [-0.01-0.01]	1.00 [0.99–1.01]	0.66
COVID-19 symptoms duration (days)	0.00 [-0.03-0.04]	1.00 [0.97–1.04]	0.88
Abbott quantitative SARS-CoV-2 Ab test (100 BAU/ml)	0.32 [0.22–0.43]	1.38 [1.25–1.53]	< 0.001
Vaccination	1.73 [0.56–2.9]	5.66 [1.76–18.2]	0.004

Notes: Model 1 was trained using data from 491 donors and its performance was evaluated on 211 donors. Model 2 was trained using data from 590 donors and its performance was evaluated on 254 donors. Model 3 was trained using data from 590 donors and its performance was evaluated on 254 donors. Model 4 was trained using data from 420 donors and its performance was evaluated on 180 donors

## Discussion

Several authors tried to find the best recruitment strategy and predictors of high antibody levels needed for improving the supply of high-quality CCP from the donors. Prudente et al. stated that among 102 individuals, the ones with a longer time interval between symptom onset and sample collection, who had been hospitalized and were above 35 years old, presented with stronger antibody response [36]. Similarly, Mehew et al. found in 29,585 CCP donors that older male donors who had been hospitalized with COVID-19 were most likely to harbor high levels of antibodies [37]. Yang et al. suggest that SARS-CoV-2 viral specific antibody response profiles are distinct in different age groups [38]. Vinkenoog et al. found that in 2,082 convalescent donors six symptoms (dry cough, fatigue, diarrhea, fever, dyspnea, and muscle weakness) predicted higher IgG concentrations [39]. Our data also shows higher neutralization antibodies in unvaccinated plasma donors (see Table 2), if they were older, had greater body weight and body mass index, and if they had higher body temperature during the infection, higher number of days with fever, cough, anosmia, and dyspnea. While obesity is a well-established independent risk factor for developing severe COVID-19, the effect of obesity on neutralizing antibody is not entirely clear with reports of positive as well as negative correlation between body weight/BMI and SARS-CoV-2 antibodies [40, 41]. The aim of our analysis was not to investigate this relationship in great detail, but it would be interesting to explore in greater detail the role of body weight on the immune response, in particular in younger patients.

We checked whether serological titer alone could represent an excellent predictive factor. Similar to previous studies [42–44], we found higher NAb titers in older male patients with higher BMI, longer-lasting fever, and higher body temperature. Our search for an optimal prediction model showed that the most crucial predictor of a suitable CCP donor was the result of the serological Abbott Quant test. In contrast, donor demographics, clinical signs, or the time of donation were not that important, and adding these variables into our logistic regression model only minimally improved its predictive power. Even a model containing the two most significant individual predictors of high NAb titers (serological titer and vaccination status) did not improve the predictive power beyond a simple model containing only the serological titer as the variable. This is due to the fact that a vast majority of the vaccinated subjects also had high serological titer values as well as high NAb values, therefore the vaccination information is already contained in the serological titer itself. Moreover, as long as the donor had a titer of anti-SARS-CoV-2 IgG above 850 BAU/ml, the probability of a high NAb titer was high (probability of 0.8). This finding suggests that measuring only SARS-CoV-2 IgG antibody concentration is sufficient to predict whether a CCP donor will have a high NT titer. This also leads to the conclusion that the laborious NT that is currently considered the gold standard can be supplemented by surrogate serological quantitative assays, which was also proved by other authors [45–52]. On the other hand, several other authors claim that commercialized serological tests, including those targeting the RBD, cannot substitute for NT assay functionality [53, 54].

In our case, we used three different serological methods (Wantai semi-quantitative SARS-CoV-2 Ab test, Abbott semi-quantitative SARS-CoV-2 Ab test, and Abbott quantitative SARS-CoV-2 Ab test) to measure anti-SARS-CoV-2 binding antibodies. The methods exhibited various abilities to separate the samples into the low and high NAb titer groups. The median was significantly different between high and low NAb titer groups for all three tests. For the Wantai test, the difference between groups was only around 10%, and the overlap in the values measured in the low and high NAb titer groups was significant. The difference between groups was approximately 60% for the semi-quantitative Abbott test. Both tests were not very useful for predicting high or low NAb titer. The quantitative Abbott test was much more helpful since the difference between groups was around 500%. Also, it is automated, easy to perform, and represents a helpful tool for providing CCP units with clinically relevant antibody titers.

In the Emergency Use Authorization (EUA), from December 2021, the FDA states that the use of CCP should be limited to units with high titers of anti-SARS-CoV-2 antibodies and that the testing criteria used in the qualification of CCP should be revised to better assure high neutralization titers in CCP. In the case of Abbott quantitative tests, previous qualifying values of ≥ 840 AU/mL are increased to ≥ 1280 AU/mL in the revised qualifying result (i.e. ≥ 119 BAU/ml to ≥ 181 BAU/ml, respectively) [55]. In our particular case, the median titers in the low and high titer groups were 192 BAU/mL, and 1123 BAU/mL, respectively (see Table S1).

The main advantage of our study is its large, representative sample size covering the whole country. However, the main limitation of our study is that the SARS-CoV-2 variant Abs specificities were limited to early variants only. The samples were collected when alpha and beta were prevalent variants. The second limitation of the study is the lack of other clinical data, such as extensive clinical parameters describing the pulmonary, immune, biochemical, and prothrombotic status during the COVID-19 infection, which might improve the prediction in similar models. Since our donors were non-hospitalized and only suffered from mild disease, such data could not be captured.

There remain several unsolved questions about whether, in the light of COVID-19 vaccination, the rise of new SARS-CoV-2 virus variants of concern (VOC), and upcoming monoclonal antibody therapies, CCP therapy is losing its potential value as a bridge therapy of the pandemic. We agree that CCP remains an option for the treatment of COVID-19 patients with impaired humoral immunity and that it could play its role as an affordable and easily accessible therapeutic and prophylactic option, especially in middle- and low-income countries [24, 25]. Besides, the national blood transfusion services may continue collecting VOC-specific CCP for the deliberate production of hyperimmune SARS-CoV-2 gamma globulins with distinct variant specificities. Some SARS-CoV-2 VOC may be less susceptible to neutralization by CCP, vaccine-elicited plasma/sera, or SARS-CoV-2 monoclonal antibodies than the earlier SARS-CoV-2 strains. In a population with a newer viral variant, locally collected CCP units will also contain neutralizing antibodies against the local variant and could be used therapeutically.

## Conclusion

The quantitative serological determination of anti-SARS-CoV-2 antibodies proved to be a sufficient predictor of high NAb titers, and adding additional demographic parameters did not improve the sensitivity and specificity of our model. Abbot Quant test for detecting anti-SARS-CoV-2 antibodies proved to be highly sensitive and specific for detecting the early SARS-CoV-2 variants and proved to be a surrogate for NT in collecting CCP units with clinically relevant antibody titers. Its value will have to be tested again in the coming months when the new waves of SARS-CoV-2 variants will emerge.

## Electronic supplementary material

Below is the link to the electronic supplementary material.

Supplementary Material 1

Supplementary Material 2

## Data Availability

The datasets generated and/or analysed during the current study are not publicly available but are available from the corresponding author on reasonable request.
