# Supplementary material for: Simple prediction of COVID-19 convalescent plasma units with high levels of neutralization antibodies
Source: Virol J. 2023 Mar 27;20:53. doi: 10.1186/s12985-023-02007-0 (PMC10042109; doi:10.1186/s12985-023-02007-0)
Supplement: Supplementary file 1 — Supplementary Material 1 [file 12985_2023_2007_MOESM1_ESM.docx]

**Table S1. The differences in plasma donor's characteristics between low and high NT titer groups**

|  | **Low NT titer <1:160)** | **High NT titer (≥1:160)** | **p-value** |
| --- | --- | --- | --- |
| **Demographic parameters** | | | |
| Gender - female | 264 (33.9%) (N=779) | 136 (26.0%) (N=523) | 0.003 |
| Age (years) | 42.5±0.4 (N=779) | 46.1±0.5 (N=523) | <0.001 |
| Body weight (kg) | 85.3±0.7 (N=650) | 88.7±0.8 (N=411) | <0.001 |
| Height (cm) | 175.9±0.3 (N=647) | 177.0±0.4 (N=408) | 0.058 |
| Body mass index (kg/m2) | 27.4±0.2 (N=646) | 28.3±0.2 (N=407) | 0.002 |
| **Blood groups and total IgG** | | | |
| Blood group 0 | 258 (33.1%) (N=779) | 158 (30.2%) (N=523) | 0.276 |
| Blood group A | 319 (40.9%) (N=779) | 239 (45.7%) (N=523) | 0.098 |
| Blood group B | 136 (17.5%) (N=779) | 88 (16.8%) (N=523) | 0.822 |
| Blood group AB | 66 (8.5%) (N=779) | 38 (7.3%) (N=523) | 0.466 |
| Rh(D) factor | 578 (81.8%) (N=707) | 354 (80.3%) (N=441) | 0.535 |
| Total IgG (AU/ml) | 10.4 [9.1-11.9] (N=745) | 10.3 [9.0-11.8] (N=502) | 0.407 |
| **Serological testing** | | | |
| Wantai semi-quantitative SARS-CoV-2 Ab test (index S/C)* | 17.9 [11.2-19.3] (N=233) | 19.6 [18.6-20.5] (N=52) | <0.001 |
| Abbott semi-quantitative SARS-CoV-2 Ab test (index S/C)* | 4.32 [2.62-6.0] (N=455) | 6.94 [5.62-7.88] (N=114) | <0.001 |
| Abbott quantitative SARS-CoV-2 Ab test (BAU/ml) | 192 [143-305] (N=398) | 1123 [446-2808] (N=446) | <0.001 |
| Neutralization test (titer) | 31.5 [30.6-32.4] (N=779) | 468.1 [451.2-485.7] (N=523) | <0.001 |
| **Timeline** | | | |
| Days after start of COVID-19 symptoms | 54.0 [41.0-80.0] (N=724) | 105.0 [49.0-190.0] (N=471) | <0.001 |
| 0-60 days after start of COVID-19 symptoms | 10.7±0.41 (N=410) | 11.72±0.54 (N=334) | 0.128 |
| 60-120 days after start of COVID-19 symptoms | 420 (58.9%) (N=713) | 158 (46.6%) (N=339) | <0.001 |
| 120-180 days after start of COVID-19 symptoms | 223 (31.3%) (N=713) | 93 (27.4%) (N=339) | 0.221 |
| **Symptoms** | | | |
| Hospitalization | 4 (1.0%) (N=335) | 1 (1.0%) (N=122) | 1.000 |
| Fever | 191 (58.0%) (N=329) | 88 (71.0%) (N=124) | 0.013 |
| Maximum body temperature (°C) | 38.0 [37.5-38.5] (N=469) | 38.0 [37.6-38.8] (N=310) | 0.046 |
| Number of days with fever | 3.2±0.2 (N=571) | 3.8±0.2 (N=433) | 0.036 |
| Cough | 106 (32.0%) (N=329) | 60 (48.0%) (N=124) | 0.002 |
| Anosmia | 209 (63.0%) (N=330) | 58 (47.0%) (N=124) | 0.002 |
| Myalgia | 146 (44.0%) (N=330) | 60 (48.0%) (N=124) | 0.460 |
| Dyspnea | 34 (10.0%) (N=329) | 25 (20.0%) (N=123) | 0.007 |
| Fatigue | 168 (51.0%) (N=329) | 63 (51.0%) (N=124) | 1.000 |
| Headache | 108 (33.0%) (N=327) | 36 (29.0%) (N=123) | 0.497 |
| **Vaccination** | | | |
| Vaccinated | 13 (1.7%) (N=779) | 222 (42.4%) (N=523) | <0.001 |
| Days after COVID-19 vaccination | 64.0 [35.5-86.5] (N=7) | 30.0 [18.0-47.8] (N=106) | 0.012 |

Notes: All data was not available for every plasma donor. The N represents the total number of samples for which the data was available for a particular parameter. *Index S/C - signal/cut-off index
