# Supplementary material for: Simple prediction of COVID-19 convalescent plasma units with high levels of neutralization antibodies
Source: Virol J. 2023 Mar 27;20:53. doi: 10.1186/s12985-023-02007-0 (PMC10042109; doi:10.1186/s12985-023-02007-0)
Supplement: Supplementary file 2 — Supplementary Material 2 [file 12985_2023_2007_MOESM2_ESM.docx]

**Table S2. The differences in vaccinated plasma donor's characteristics between low and high NT titer groups**

|  | **Low NT titer (<1:160)** | **High NT titer (≥1:160)** | **p-value** |
| --- | --- | --- | --- |
| **Demographic parameters** | | | |
| Gender - female | 1 (7.7%) (N=13) | 52 (23.4%) (N=222) | 0.307 |
| Age (years) | 45.5±2.5 (N=13) | 44.2±0.7 (N=222) | 0.675 |
| Body weight (kg) | 90.5±3.0 (N=13) | 87.2±1.0 (N=212) | 0.444 |
| Height (cm) | 182.6±2.0 (N=13) | 177.9±0.6 (N=212) | 0.049 |
| Body mass index (kg/m2) | 27.2±0.8 (N=13) | 27.5±0.3 (N=211) | 0.749 |
| **Blood groups and total IgG** | | | |
| Blood group 0 | 6 (46.2%) (N=13) | 52 (23.4%) (N=222) | 0.093 |
| Blood group A | 5 (38.5%) (N=13) | 112 (50.5%) (N=222) | 0.570 |
| Blood group B | 2 (15.4%) (N=13) | 40 (18.0%) (N=222) | 1.000 |
| Blood group AB | 0 (0.0%) (N=13) | 18 (8.1%) (N=222) | <0.001 |
| Rh(D) factor | 9 (81.8%) (N=11) | 150 (80.6%) (N=186) | 1.000 |
| Total IgG (AU/ml) | 10.0 [8.7-11.1] (N=13) | 9.9 [8.6-11.3] (N=222) | 0.953 |
| **Serological testing** | | | |
| Abbott quantitative SARS-CoV-2 Ab test (BAU/ml) | 512 [307-755] (N=13) | 2243 [1172-4172] (N=222) | <0.001 |
| Neutralization test (titer) | 55.1 [48.5-62.5] (N=13) | 855.6 [821.2-891.5] (N=222) | <0.001 |
| **Vaccination** | | | |
| Vaccinated | 13 (100.0%) (N=13) | 222 (100.0%) (N=222) | 1.000 |
| Days after COVID-19 vaccination | 64.0 [35.5-86.5] (N=7) | 30.0 [18.0-47.8] (N=106) | 0.012 |

Notes: All data was not available for every plasma donor. The N represents the total number of samples for which the data was available for a particular parameter. *Index S/C - signal/cut-off index
